# Assessment of patient safety culture in primary care setting, Al-Mukala, Yemen

**DOI:** 10.1186/s12875-015-0355-1

**Published:** 2015-10-13

**Authors:** Hana H. Webair, Salwa S. Al-assani, Reema H. Al-haddad, Wafa H. Al-Shaeeb, Manal A. Bin Selm, Abdulla S. Alyamani

**Affiliations:** Family Medicine Department, Hadhramout University College of Medicine, Al-Mukala, Yemen; Ba’abood Family Medicine Centre, Ministry of Public Health and Population, Al-Mukala, Yemen; Fowa Alqadeema Primary Care Centre, Ministry of Public Health and population, Al-Mukala, Yemen

**Keywords:** Patient safety culture, Primary care, Yemen

## Abstract

**Background:**

Patient safety culture in primary care is the first step to achieve high quality health care. This study aims to provide a baseline assessment of patient safety culture in primary care settings in Al-Mukala, Yemen as a first published study from a least developed country.

**Methods:**

A survey was conducted in primary healthcare centres and units in Al-Mukala District, Yemen. A comprehensive sample from the available 16 centres was included. An Arabic version of the Medical Office Survey on Patient Safety Culture was distributed to all health workers (110). Participants were physicians, nurses and administrative staff.

**Results:**

The response rate from the participating centres was 71 %. (*N* = 78). The percent positive responses of the items is equal to the percentage of participants who answered positively. Composite scores were calculated by averaging the percent positive response on the items within a dimension. Positive safety culture was defined as 60 % or more positive responses on items or dimensions. Patient safety culture was perceived to be generally positive with the exception of the dimensions of ‘Communication openness’, ‘Work pressure and pace’ and ‘Patient care tracking/follow-up’, as the percent positive response of these dimensions were 58, 57, and 52 % respectively. Overall, positive rating on quality and patient safety were low (49 and 46 % respectively).

**Conclusions:**

Although patient safety culture in Al-Mukala primary care setting is generally positive, patient safety and quality rating were fairly low. Implementation of a safety and quality management system in Al-Mukala primary care setting are paramount. Further research is needed to confirm the applicability of the Medical Office Survey on Patient Safety Culture (MOSPSC) for Al-Mukala primary care.

## Background

Quality and safety are the vital goals for all health care organizations. Patient safety means the extent to which patients are protected from avoidable harm, poor patient safety indicates that patients are not in fact adequately protected [1].

Most researchers and activities are directed to hospitals although it is well known that the majority of patients are treated and cared for in primary care facilities, especially by family doctors [2]. This is especially true in developing countries, often with significant limitations on infrastructure, as well as in procedures and standards for safe practices [2]. Eastern Mediterranean and African Study found that unsafe care affects around 10 % of patients, most those incidents were preventable [3].

It goes without saying that patient safety is a challenge against primary care success [4]. Actually, the amount of medical errors in primary care has been found to be difficult to estimate, as it depends on the accuracy of recording and incidents standardization so very little is known about these errors [5]. It has been identified that a significant proportion of safety incidents caught in hospitals had originated in the earlier levels of care [2].

As a result, the World Health Organization (WHO) Patient Safety Program has initiated the “Safer Primary Care” project. It focuses on risk exposures, harms which are preventable, and how to protect patients at primary care level [6].

In order to enhance primary care safety, the National Patient Safety Agency developed a best practice guide that describes how to “build a safety culture” as the first of the seven key steps for primary care organizations to protect the patients they care for [7]. However, undertaking a baseline assessment of patient safety culture of the organization is the first step to start with in building safety culture [2].

A true safety culture is one in which every person in the organization recognizes their responsibilities in regard to patient safety and works to improve the care they deliver. In addition to a recognition that mistakes and incidents can happen, and that health care is not without its risks [7].

Consensus has emerged among patient safety experts that cultural attributes such as leadership support, teamwork, communication, and fair and just culture principles remain central to ensuring patient safety in health care organizations [8].

Measuring the patient safety culture helps organizations to detect areas for improvement and monitor changes over time [9]. A number of tools have been used in various healthcare settings—most of them have been designed in developed countries [9, 10].

However, culture and other human factors have influences on patient safety so these factors should be considered whenever safety culture measurement tools are applied in different social settings [10, 11].

There are few published studies on patient safety culture in primary care and most of them are in developed countries [12–20]. There is only one published study assessing primary care patient safety culture in an Arabic population (Kuwait) [19], and two studies in the Eastern Mediterranean Region (EMRO) [15, 19]. Ghobashi et al. assessed patient safety culture in Kuwait primary care centers and found that the mean score for positive perception of patient safety culture dimensions was 56 % [19]. It was slightly higher in Iranian health centers (57 %) [15]. Unfortunately, primary care patient safety culture has not been assessed in least developed countries.

The current study aims to provide a baseline assessment of patient safety culture in primary care settings in AL-Mukala, Yemen. It can provide insight into areas for improvement to guide future changes.

## Methods

### Study setting

This study has been conducted in Al-Mukala District’s primary health care centers and units (PHCCs). Al-Mukala is the capital of Hadhramout, Yemen. There were 16 health centers and units in Al-Mukala District at the time of study. All of them contain at least one outpatient clinic. Some of these centers contains more clinics such as general practice, gynecology and nutrition clinics. All clinics in the center share the same administrative staff and most non-clinical support staff. Most of the managers are care providers. The majority of these centers are small buildings with scarce resources. Most of them lack quality and safety systems. An information exchange system is not available so communication with other settings occurs informally. There is no information system or medical records in most centers. The total number of staff in each center varies from 3 to16.

### Design and sampling

A survey was conducted in the period between June to December 2013. The sample was comprehensive which included all providers and non-care providers in the 16 PHCCs. The sample included physicians, nurses including medical assistants and midwives, and non-clinical staff (non-care providers). The questionnaires were distributed to 110 providers and non-care providers who were available at the time of study. Those who spent less than a month in the center were excluded.

### Data collection tool

The current study used the Medical Office Survey on Patient Safety Culture (MOSPSC) which is a validated tool sponsored by the Agency of Healthcare Research and Quality (AHRQ) for medical offices [21]. It has sound psychometric properties and was released first in 2009 [22]. Al-Mukala’s PHCCs met the criteria of AHRQ for medical offices so were eligible for using this survey tool. The criteria are that the medical office should be an outpatient facility in one geographic place. Providers in the medical office should share some or all administrative staff, and clinical support staff. Administration of MOSPSC is restricted to offices with at least three providers. Providers are physicians, and other providers licensed to diagnose health problems, treat patients, and prescribe drugs [21].

The medical office survey tool composed of two overall safety outcomes and twelve dimensions. It has been adapted and validated for use in primary healthcare settings in Spain, it has been found to be useful and recommended for international comparison [16]. It has been translated into Arabic by a translator who has experience in patient safety research, then back-translated to test translation accuracy. The translation was then reviewed by six professional experts from the primary care and patient safety fields. Lastly, the questionnaire was piloted with five health workers to make sure the questions were understood and not unpleasant.

Modification was done in light of a pilot study and the last two dimensions of MOSPSC (information exchange with other settings, and patient safety and quality issues) were deleted because of the high non response rate and non-applicability. So the current study used the following survey measures; first: two overall patient safety outcomes (6 items). i.e. overall ratings on quality and overall rating on patient safety, second: ten dimensions of culture related to patient safety (38 items): teamwork, patient care tracking/follow up, organizational learning, overall perceptions of patient safety and quality, staff training, owner managing partner/leadership support for patient safety, communication about error, communication openness, office process and standardization, and work pressure and pace [21]. The 10 dimensions’ reliability expressed as Cronbach’s alpha for the AHRQ data from more than 200 medical offices ranged from 0.75 to 0.83 [21]. whereas for the data in this research, the Cronbach’s alpha ranged from 0.20 to 0.70 (Table 1), much lower than the AHRQ data, which inferred that the consistency of the responses on each survey item for the data in this study is very low if compared with the AHRQ data.

**Table 1:** Item-level result for Al-Mukala primary care centers (Yemen, *N* = 78) and U.S. medical offices (*N* = 27,103)

Survey Items By Patient Safety Culture Dimensions	% positive response	
	PHCCs^a^	Benchmark^b^
1. Teamwork (Cronbach’s alpha = 0.468)		
1. When someone in this office gets really busy, others help out. C1	97	87
2. In this office, there is a good working relationship between staff and providers. C2	97	89
3. In this office, we treat each other with respect. C5	96	84
4. This office emphasizes teamwork in taking care of patients C13	94	86
2. Patient Care Tracking/Follow-up (Cronbach’s alpha = 0.289)		
1. This office reminds patients when they need to schedule an appointment for preventive or routine care. D3	60	87
2. This office documents how well our chronic-care patients follow their treatment plans. D5	55	80
3. Our office follows up when we do not receive a report we are expecting from an outside provider. D6	26	88
4. This office follows up with patients who need monitoring. D9	68	91
3. Organizational Learning (Cronbach’s alpha = 0.402)		
1. When there is a problem m our office, we see if we need to change the way we do things. F1	86	85
2. This office is good at changing office processes to make sure the same problems don’t happen again. F5	64	80
3. After this office makes changes to improve the patient care process, we check to see if the changes worked. F7	100	76
4. Overall Perceptions of Patient Safety and Quality (Cronbach’s alpha = 0.259)		
1. Our office processes are good at preventing mistakes that could affect patients. F2	87	86
2. Mistakes happen more than they should in this office. F3^c^	98	80
3. It is just by chance that we don’t make more mistakes that affect our patients. F4^c^	85	81
4. In this office, getting more work done is more important than quality of care. F6^c^	37	74
5. Staff Training (Cronbach’s alpha = 0.399)		
1. This office trains staff when new processes are put into place. C4	57	78
2. This office makes sure staff get the on-the-job training they need. C7	74	77
3. Staff in this office are asked to do tasks they haven’t been trained to do. C10^c^	74	70
6. Owner/Managing Partner/Leadership Support for Patient Safety (Cronbach’s alpha = 0.697)		
1. They aren’t investing enough resources to improve the quality of care in this office. E1^c^	50	52
2. They overlook patient care mistakes that happen over and over. E2^c^	69	S3
3. They place a high priority on improving patient care processes. E3	78	82
4. They make decisions too often based on what is best for the office rather than what is best for patients. E4^c^	59	62
7. Communication About Error (Cronbach’s alpha = 0.197)		
1. Staff feel like their mistakes are held against them. D7^c^	67	61
2. Providers and staff talk openly about office problems. D8	79	61
3. In this office, we discuss ways to prevent errors from happening again. D11	74	82
4. Staff are willing to report mistakes they observe in this office. D12	48	76
8. Communication Openness (Cronbach's alpha = 0.632)		
1. Providers in this office are open to staff ideas about how to improve office processes. D1	53	70
2. Staff are encouraged to express alternative viewpoints in this office. D2	48	71
3. Staff are afraid to ask questions when something does not seem right. D4^c^	72	73
4. It is difficult to voice disagreement in this office. D10^c^	61	57
9. Office Processes and Standardization (Cronbach’s alpha = 0.365)		
1. This office is more disorganized than it should be. C8^c^	46	66
2. We have good procedures for checking that work m this office was done correctly. C9	73	73
3. We have problems with workflow in this office. C12^c^	59	54
4. Staff in this office follow standardized processes to get tasks done. C15	81	82
10. Work Pressure and Pace (Cronbach’s alpha = 0.404)		
1. In this office, we often feel rushed when taking care of patients. C3^c^	67	37
2. We have too many patients for the number of providers in this office. C6^c^	58	49
3. We have enough staff to handle our patient load. C11	49	51
4. This office has too many patients to be able to handle everything effectively. C14^c^	55	62

^a^PHCCs: Primary Health Care Centers

^b^Benchmaik: is data obtained from 935 U.S. medical offices of different specialties, most categorized as Family Practice (391 offices) [23]

^c^Negatively worded items

If the following six items are deleted, the reliability will become better (range from 0.23 to 0.81) with only one dimension reliability below 0.40. These items are C3, C9, C10, D3, D8, and F6. To justify the validity of using the MSOPSC on assessing patient safety culture in Al-Mukala primary care setting, we planned to use the confirmatory factor analysis (CFA) but it did not meet the test assumptions because of the inadequacy of the sample size.

### Data collection method

The data were collected by paper-based self-administered questionnaires. Questionnaires were distributed to the 16 health centers and units by the researchers and health workers. There were two surveys one week apart to maximize the response rate as recommended by the questionnaire developers [21]. The second survey excluded participants who had filled out the questionnaire during the first survey. Each health center's or unit's questionnaires were uniqely identified.. After receiving the completed questionnaires, surveys were examined for completeness. Surveys where the respondent gave the exact same answer to all the questions were omitted as well as blank ones [21]. After removing incomplete questionnaires, a total of 78 respondents from 16 PHCCs provided completed surveys (17 physicians, 46 nurses, and 15 non-care providers). Therefore, the final response rate for the survey was 71 %.

### Data analysis

**Fig. 1:**
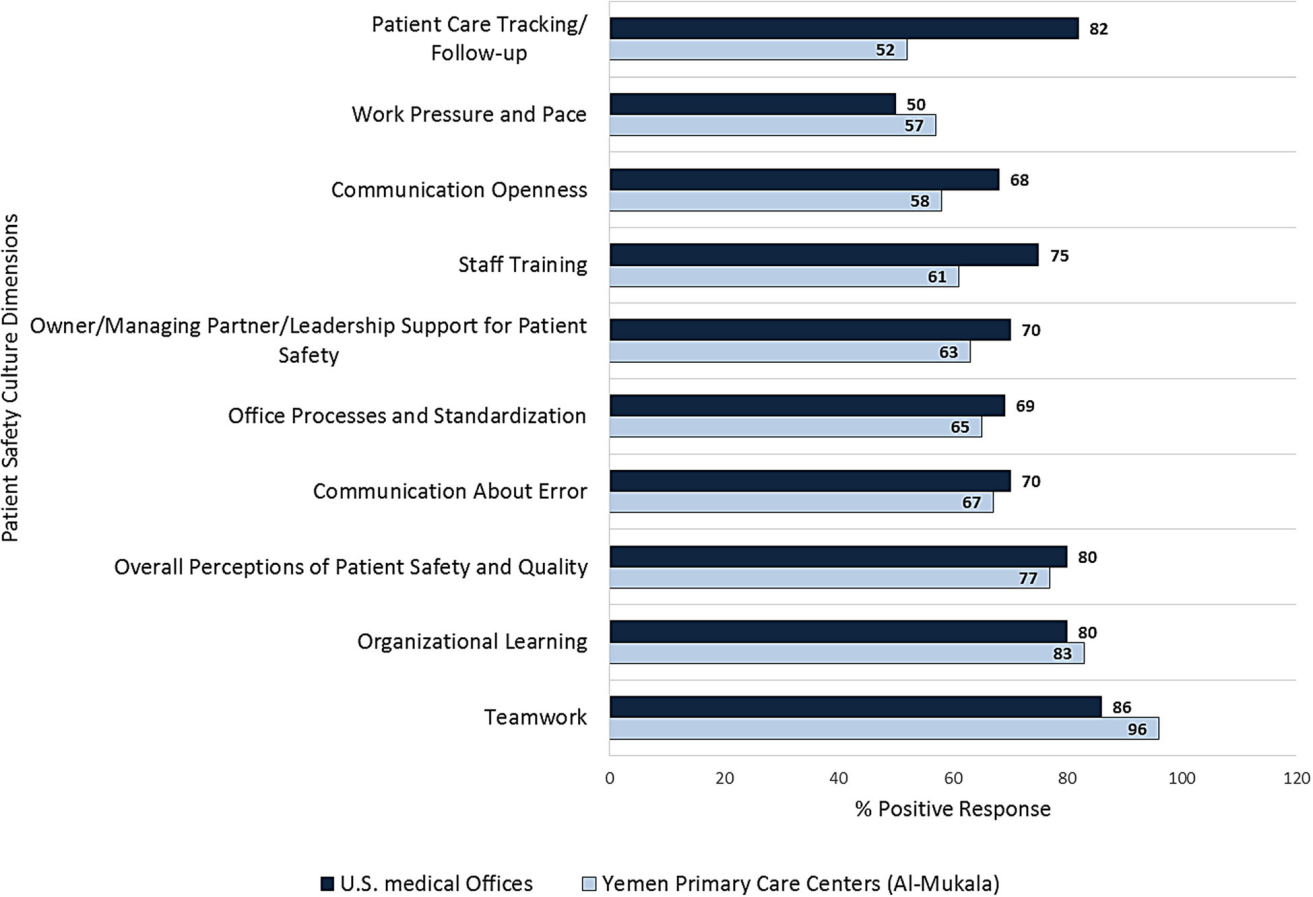
Comparison of composite-level positive scores for Al-Mukala. (Yemen) primary care centers with benchmark

**Fig. 2:**
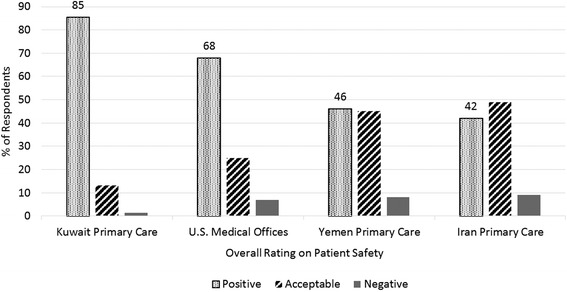
Comparison of overall patient safety culture grade for Al-Mukala. (Yemen) primary care centers and U.S. medical offices, Kuwaiti and Iranian primary care centers

The data were entered and analyzed by the researchers using the Premier customized data tool [21] and IBM SPSS Statistics 20. *Calculation of percent positive responses:* Item percent positive responses for each positively worded question is equal to the percentage of positive responses i.e. ‘strongly agree’/, ‘agree’, or ‘excellent’/‘very good’. For example, for the item “We have enough staff to handle our patient load,” if 30 % of respondents within a medical office responded “Strongly agree” and 40 % responded “Agree”, the item-level percent positive response would be 30 % + 40 % = 70 %. Likewise, for each negatively worded item, the percentage of negative responses was calculated. For example, for the item “Mistakes happen more than they should in this office,” if 60 % of respondents within a medical office responded “strongly disagree” and 20 % responded “disagree”, the item-level percent positive response would be 80 % (i.e., 80 % of respondents do not believe mistakes happen more than they should in this office). Composite scores were calculated by averaging the percent positive response on the items within a dimension. For example, for a four-item composite, if the item-level percent positive responses were 40, 50, 60 and 50 %, the medical office’s composite-level percent positive response would be the average of these four percentages, or 50 % positive. Patient safety strengths are items/dimensions with 75 or more percent positive response [21]. The cutoff percentage for areas needing improvement is less than 60 % positive response. *Univariate analysis:* descriptive statistics for the participants’ characteristics as well as patient safety outcomes were calculated. *Bivariate analysis:* The PHCCs items and composite score were compared against the results from 935 United States (U.S.) medical offices of different specialties (benchmark score), with most categorized as Family Practice (391 offices) as seen in Table 1 & Fig. 1. The 2014 database consists of data from 27,103 respondents, a range of 5 to 725 completed surveys were submitted per medical office, and the average response rate was 64 % [23]. Comparison with results from regional surveys was impossible because none of them used the same tool. The overall rating of patient safety was compared against results from Kuwait, Iran and U.S. medical offices (Fig. 2).

### Ethical considerations

The study protocol has been approved by the department of Family Medicine, Hadhramout University College of Medicine. Permission letters were sent to the managers of the health centers and verbal informed consents were obtained from all the respondents for agreement about participation.

## Results

### Demographic data

A total of 78 healthcare staff provided survey feedback (a response rate of 71 %). Fifty-six (72 %) of the participants were females. The majority, 63 (81 %) of them, were providers. Seventeen of respondents (22 %) were physicians and 46(59 %) were nurses. Most of them had a diploma (67 of them (86 %)). Around half of respondents had patient safety education 40(51 %). More than half of the healthcare staff had work experience of 3 years or more in the current health center (44 of them (56 %)). Most had work duties of less than 33 hours per week (83 %) (Table 2).

**Table 2:** Demographic characteristics of respondents in Al-Mukala (Yemen) primary care centers

Variable		No (%)
Gender	Male	22(28.21)
	Female	56(7 *l.79)*
Qualification	Diploma	67(85.90)
	Bachelor or master	11(14.10)
Job position	Care providers	63(80.77)
	Non-care providers	15(19.23)
Patient safety education	Yes	40(51.28)
	No	38(48.72)
Duration of work in the health center (Year)	<1	17(21.79)
	1 - < 3	17(21.79)
	3- < 6	16(20.51)
	6- <11	12(15.38)
	11 or more	16(20.51)
Work hours per week	<16	18(23.08)
	16 - < 25	30(38.46)
	25–33	17(21.79)
	3 3 or more	13(16.67)
Total		78(100)

### Patient safety culture dimensions

The average positive responses for all dimensions was 67 %. Fig. 1 demonstrates the percentage of positive responses in the ten dimensions in the PHCCs. It was highest for ‘teamwork’ (96 %), and ‘Organizational learning’ (83 %) while lowest for ‘Work pressure and pace’ (57 %) and ‘Patient care tracking/follow-up’ (52 %). In comparison with the benchmark average score obtained from 935 medical offices in U.S., the score for ‘teamwork’ was lower in medical offices (86 %), than in PHCCs. On the other hand, the positive score for ‘Patient Care Tracking/Follow-up’ was very low for PHCCs (52 %) if compared with medical offices (82 %).

### Healthcare quality and patient safety grade

The average positive rating on quality was very low (49 %) in PHCCs in contrast with medical offices (68 %) (Table 2). Patient centeredness in PHCCs had the lowest rating among all of the quality dimensions. It was assessed as very good to excellent by only 33 % of participants. Whereas equitability had the highest positive rating (81 %) in both PHCCs and U.S. medical offices (82 %) (Table 3). Concerning patient safety, positive overall rating (excellent and very good) on patient safety in Al-Mukala PHCCs (46 %) was less than in Kuwaiti PHCCs and U.S. medical offices as shown in Fig. 2.

**Table 3:** Overall rating on quality; comparative results for Al-Mukala primary healthcare centers (Yemen, *N* = 78) and U.S. medical offices (*N* = 27,103)

Rating	Excellent % PHCCs(MO)^b^	Very good % PHCCs (MO)	Good % PHCCs (MO)	Fair % PHCCs (MO)	Poor % PHCCs (MO)
Quality dimension^a^					
i. Patient centeredness	8(36)	25(36)	32(23)	19(5)	16(1)
ii. Effective	17(34)	23(37)	41(25)	17(4)	1(1)
iii. Timely	12(23)	31(33)	32(28)	21(12)	4(4)
iv. Efficient	22(26)	24(35)	43(28)	7(8)	4(2)
v. Equitable	44(55)	37(27)	13(14)	4(3)	1(1)

^a^Quality dimension items are: i. is responsive to individual centered patient preferences, needs, and values, ii. is based on scientific knowledge, iii. minimizes waits and potentially harmful delays, iv. ensures cost-effective care (avoids waste, overuse and misuse of services), v. provides the same quality of care to all individuals regardless gender, race, ethnicity, socioeconomic status, language …etc

^b^ *PHCCs: * AL-Mukala primary healthcare centers (Yemen), *MO:* U.S. medical offices

## Discussion

To our knowledge, this study is the first published that assessed PHCCs patient safety culture in Yemen and least developed countries. However, research is a priority to promote patient safety in primary care [24]. On the other hand, there are many studies conducted in developing and developed countries on patient safety culture in primary care with diversity both in the tools used and outcomes reporting. But only one published study used MOSPSC in primary care [16].

There were many areas of strengths and others with potential for improvement. Areas requiring improvement are patient care tracking/follow up, communication openness, and work pressure and pace. These areas should be focused on because positive safety culture is so important to improve patient safety in primary care [25].

The average of positive responses for all dimensions in the PHCCs was 67 % which was lower than in U.S. medical offices’ average but higher than in Turkish (47 %) [12], Iranian [15], and Kuwaiti PHCCs [19]. Our PHCCs differ from other countries by the very small size and less diversity of team members. Sample size of the above-mentioned studies ranged from 100–276, and their participants included dentists, dieticians, pharmacists, technicians, and community health workers in addition to physicians, nurses and administrative staff. Our high positive response here could be explained by the findings from the U.S medical office comparative database. It shows that the greater the number of providers, the lower average percent positive on all ten patient safety culture dimensions [23]. Members of small teams may have a more positive perception of team climate in general and work more closely together despite their different professions [26, 27].

The highest percentage of positive responses in the current study were in “teamwork” and “organizational learning” dimensions. Most Al-Mukala PHCCs are small buildings with few staff and an unsophisticated environment which are factors that encourage teamwork [24].

However, these dimensions were areas of strength in many studies regionally and internationally as in Kuwaiti PHCCs, U.S. medical offices and hospitals, as well as in Taiwanese, Lebanese, and Saudi hospitals [11, 19, 23, 28, 29].

On the other hand, the least positive response was in patient care tracking/follow up. This means that in Al-Mukala PHCCs patients are not reminded about appointments, their compliance with the treatment plan is not documented, follow up with patients who need monitoring or when reports from an outside provider are not received are lacking. In contrast, the U.S. medical offices found that patient care tracking was the second highest positive dimension [21]. Unlike the U.S. health system, AL-Mukala PHCCs are characterized by less modernization and lack of an electronic system which makes patient follow up more difficult. Information technology is very important for patient safety as it facilitates rapid tracking and follow-up of medical errors [30].

The second area for improvement in this study is inadequacy of staff and providers to handle the patient load, and the deficiency of work pace. Similarly, benchmark medical offices and many other studies conducted in primary care settings and hospitals reported inadequacy of staff and work load as areas of weakness [12, 15, 19, 23, 31]. It has been clarified methodologically that the number of PHCCs in Al- Mukala district and staff in each center are generally few which explains the reason of work pressure. Most published studies in PHCCs used a modified version of the AHRQ hospital survey that does not assess patient care tracking. In those studies, the frequency of events reported, the non-punitive response, in addition to staffing had the lowest positive responses [12, 15, 19, 31]. A very low positive response for event reporting is expected because primary care is known to lack standardized incidents registration or reporting systems [5]. Zwart et al. reported that incident reporting is actually uncommon in Dutch general practice [32]. So it is realistic to overlook this dimension in MOSPSC.

The third area of concern was that superiors in the PHCCs are not open to staff ideas, and staff are not encouraged to say alternative viewpoints or express disagreement. Communication openness was an area of concern in studies in Kuwait and Turkey [12, 19], but Iranian and Dutch PHCCs, and U.S. medical offices reported higher positivity [15, 23, 31]. The discrepancy between results regarding communication openness from different countries might be related to cultural differences especially communication styles. For example, Americans tend to be direct in communication. They value logic and linear thinking and expect people to speak frankly and in a straightforward manner [33]. However, openness in general is found to be a problem in developing countries and the Middle East [34]. Yemenis like many Eastern populations tend to be conservative in conversation and feedback, so frank criticism is usually not acceptable [35]. Disagreement and criticism against supervisors or team members are frequently interpreted as blame or as a fight against them and may lead to loss of personal relationship or career so most employees tend to avoid it.

Overall, positive rating of healthcare safety and quality in this study was low in all areas (less than 50 %) except equitability, where they were rated positive by 81 % (Table 3). This result is not surprising due to a lack of formal safety and quality management systems in our primary care centers. Our health centers’ responsiveness to individual patient preferences, needs, and values was an area of concern. Patient-centeredness in health care has been proved to have a positive impact on patient safety [36]. However, in Yemen, decisions are generally made by the superiors and work their way down, especially in public sectors [35]. So in the domain of healthcare, patients are infrequently involved in the process and their opinions and preferences are not priorities. In the same vein, Yemen has in general a slow-paced culture, delays to business and appointments are not uncommon and is not interpreted as a matter of disrespect or impoliteness. This is starting to change slowly as the pace of life is starting to become faster and faster [35]. This feature is probably reflected in healthcare quality making it untimely.

Less than half of respondents in this study gave positive overall rating of patient safety, a similar result was reported in Turkish and Iranian PHCCs [12, 15]. While in Kuwaiti PHCCs, U.S. medical offices, and hospitals as well as Lebanese and Palestinian ones, the most frequent rating was excellent to very good [19, 21, 28, 37]. Overall rating of patient safety assesses systems and clinical processes undertaken by the organization to prevent, detect, and correct problems that could endanger patients [21]. Primary care in developing countries is characterized by suboptimal infrastructure, procedures and standards for safe practices [6]. Al-Mukala PHCCs lack safety and quality systems. Some efforts are done informally to prevent harm but they are inadequate.

## Conclusions

Though patient safety culture in Al-Mukala primary care setting is positive overall, patient safety and quality rating were fairly low. The systems and clinical processes to prevent, catch, and correct problems that have the potential to affect patients are inadequate in Al-Mukala health centres. Adding to that, low quality of health care concerning patient-centeredness, effectiveness, timeliness, and efficiency. The highest percent positive responses were for ‘teamwork’ and ‘organizational learning’. Areas of potential for improvement are communication openness, patient care tracking/follow up, and work pressure and pace. Implementation of safety and quality management systems in Al-Mukala primary care setting is paramount. We recommend increasing the number of health workers per centre and finding an appropriate method for effective patient care tracking. Communication between health care providers and the staff within health centres needs to be more clear and direct in order to encourage constructive criticism and to discover mistakes and errors and how to avoid them in future. Further research is needed to ensure the applicability of the MOSPSC for Al-Mukala primary care. There were several limitations to this project. The number of health workers in Al- Mukala health centres was small which led to a small sample size. Since the majority of respondents were physicians and nurses, the results did not adequately reflect the perception of other respondent groups, so the comparison by staff position was not conducted. Another limitation relates to the low Cronbach’s alpha values for the composite scores measuring patient safety culture in Al-Mukala PHCCs. Such low scores may have resulted from the fact that some terminology may be unknown to Al- Mukala PHCCs’ staff because the concept of patient safety culture is new and because there is a lack of safety and quality management systems. Testing the validity of MOSPSC was impossible due to an inadequate sample.

## Abbreviations

AHRQ: Agency of Healthcare Research and Quality
EMRO: Eastern Mediterranean Region
IOM: International institute of medicine
MOSPSC: Medical Office Survey on Patient Safety Culture
PHCCs: Primary health care centers
SPSS: Statistical product and service solutions
U.S.: United States
WHO: World Health Organization
